# Case Report: Colchicine Toxicokinetic Analysis in a Poisoned Child Requiring Extracorporeal Life Support

**DOI:** 10.3389/fped.2021.658347

**Published:** 2021-04-07

**Authors:** Maria Pérez Marín, Sylvain Prod'hom, Suzanne Francesca de Villiers, Thomas Ferry, Vivianne Amiet, Julia Natterer, Maria-Helena Perez, Thierry Buclin, Haithem Chtioui, David Longchamp

**Affiliations:** ^1^Pediatric Intensive Care Unit, Lausanne University Hospital and University of Lausanne, Lausanne, Switzerland; ^2^Service of Clinical Pharmacology, Lausanne University Hospital and University of Lausanne, Lausanne, Switzerland

**Keywords:** colchicine poisoning, ECMO, toxicokinetics, pediatric, case report

## Abstract

Colchicine poisoning is associated with a poor prognosis, especially when leading to shock and multi-organ failure, and management is limited to supportive care, including multiple-dose activated charcoal. At therapeutic concentrations, colchicine elimination occurs mainly through hepatic metabolism and involves an enterohepatic circulation, with a small contribution of renal elimination (10–30%). Colchicine toxicokinetics is however rarely described, especially in children. We present the case of a 4-year-old patient who survived a severe iatrogenic colchicine intoxication with a dose of 0.5 mg/kg. She developed multi-organ failure and shock, but recovered after receiving aggressive resuscitation, including extracorporeal life support. Close monitoring of colchicine blood levels showed a plateau for 6 days, indicating impeded elimination resulting from liver failure. We observed no significant clearance from renal replacement therapy, nor activated charcoal, during this period. Extracorporeal life support may play a supportive role in the management of severe colchicine poisoning. However, extracorporeal techniques do not seem to improve colchicine elimination.

## Introduction

Colchicine is a plant-derived inhibitor of microtubule polymerization. Its off-label use in various inflammatory conditions include pericarditis. The narrow therapeutic index makes it a potentially hazardous drug. The pharmacokinetics in the context of colchicine overdose have been poorly studied and data about pediatric cases are scarce. Colchicine metabolism and elimination may be significantly impacted by organ dysfunction, thus contributing to long-lasting drug overexposure. Extracorporeal elimination therapies have questionable efficacy on colchicine clearance.

We describe here the case of a 4-year-old patient who survived a single, accidental, severe colchicine overdose. Extracorporeal life support was successfully used to recover from cardiovascular collapse. Thorough follow-up of colchicine blood levels was documented, allowing toxicokinetic analysis.

## Case Description

A 4-year-old girl, weighing 13 kg, had surgical correction of a congenital atrioventricular septal defect, 1 month previously. Because of a persistent postoperative pericardial effusion, colchicine treatment was prescribed. Due to a misunderstanding, a prescription error occurred and she received a single accidental overdose of 0.5 mg/kg (total: 7 mg) instead of the intended 0.5 mg/dose. 8 to 10 h after ingestion, the patient developed vomiting, diarrhea, tachycardia, tachypnea and somnolence. In the meantime, the overdose was identified and the patient was transferred to the pediatric intensive care unit (PICU).

Activated charcoal was administered and continued until day 8 after ingestion, up to a total of 186 grams. Due to progressive sleepiness, she was intubated (18 h post-ingestion). Immediately after induction (sevoflurane) for intubation and central venous catheter placement, she developed severe hypotension (non-invasive blood pressure 39/21 mmHg) and hypoxemia (SpO_2_ 63%) requiring fluid boluses and vasoactive support. Echocardiography showed massive dilatation of the right ventricle (RV) and consecutive severe tricuspid regurgitation with increased estimated RV pressure. RV function improved with nitric oxide treatment. However, cardiogenic shock persisted despite fluid resuscitation, catecholamine (noradrenaline, adrenaline) and vasopressin treatment, and percutaneous drainage of the previously diagnosed pericardial effusion. RV failure persisted, while the left ventricle suffered moderate systolic dysfunction and a grade I mitral regurgitation. Central veno-arterial extracorporeal membrane oxygenation (VA ECMO) was initiated, providing a flow rate of 2.6 L/min/m^2^ and enabling rapid weaning of adrenaline and vasopressin. Once cardiac function improved, with recovery of the dilated RV and improvement of biventricular contractility, ECMO flow was gradually decreased over 3 days, for a total of a seven days uneventful support. A 48-h levosimendan infusion was administered to prepare ECMO weaning.

In addition to heart failure, she developed multi-organ failure with hepatic, renal and bone marrow involvement. On day 2, she developed anuria due to acute tubular necrosis. Continuous renal replacement therapy (hemofiltration) was applied from day 2 to 16. The blood flow rate was 100 ml/min, with an extraction depending on the patient's fluid balance. She developed hypertension in relation to kidney failure, apparent after stopping sedation. On day 1, severe liver failure occurred with cytolysis and coagulation disorder, requiring several administrations of vitamin K, fibrinogen and fresh frozen plasma, with gradual improvement from day 4. Liver function monitoring showed a peak value of bilirubin on day 5 (total bilirubin 35 μmol/l, direct bilirubin 25 μmol/l), the lowest value of factor V on day 2 (16%) with normalization on day 6 and the peak value of ammonium on day 2 (87 μmol/L) with normalization on day 4. An initial leukocytosis on day 1 (14.9 G/L) was followed by subsequent bone marrow failure with leukopenia (nadir leukocyte count 1.3 G/l on day 5) and thrombocytopenia (nadir platelet count 9 G/l on day 11). Multiple platelet and red blood cell transfusions were required. She received granulocyte colony-stimulating factor (G-CSF) from day 4 to 6, causing rebound leukocytosis from day 8 onwards (maximum 48.3 G/l on day 10) (Supplementary Figure 1). A blood smear on day 13 showed a good recovery of the marrow. Because of liver failure, myelosuppression and colonization by Extended Spectrum Beta-Lactamase (ESBL) producing Enterobacteriaceae, she received meropenem from day 2 to 10. The respiratory course was slowly favorable, allowing extubation on day 19 with relay by non-invasive ventilation until day 22. An EEG performed on day 15 showed an overall slowdown with conserved reactivity and no epileptiform activity. After extubation, she had global weakness, consistent with a critical illness polyneuromyopathy likely superimposed to colchicine peripheral neurological toxicity. The weakness improved over weeks with physiotherapy. Alopecia was noted from day 19 onwards. She was discharged from the PICU on day 27, and from the hospital on day 63 with full recovery.

**Figure 1:**
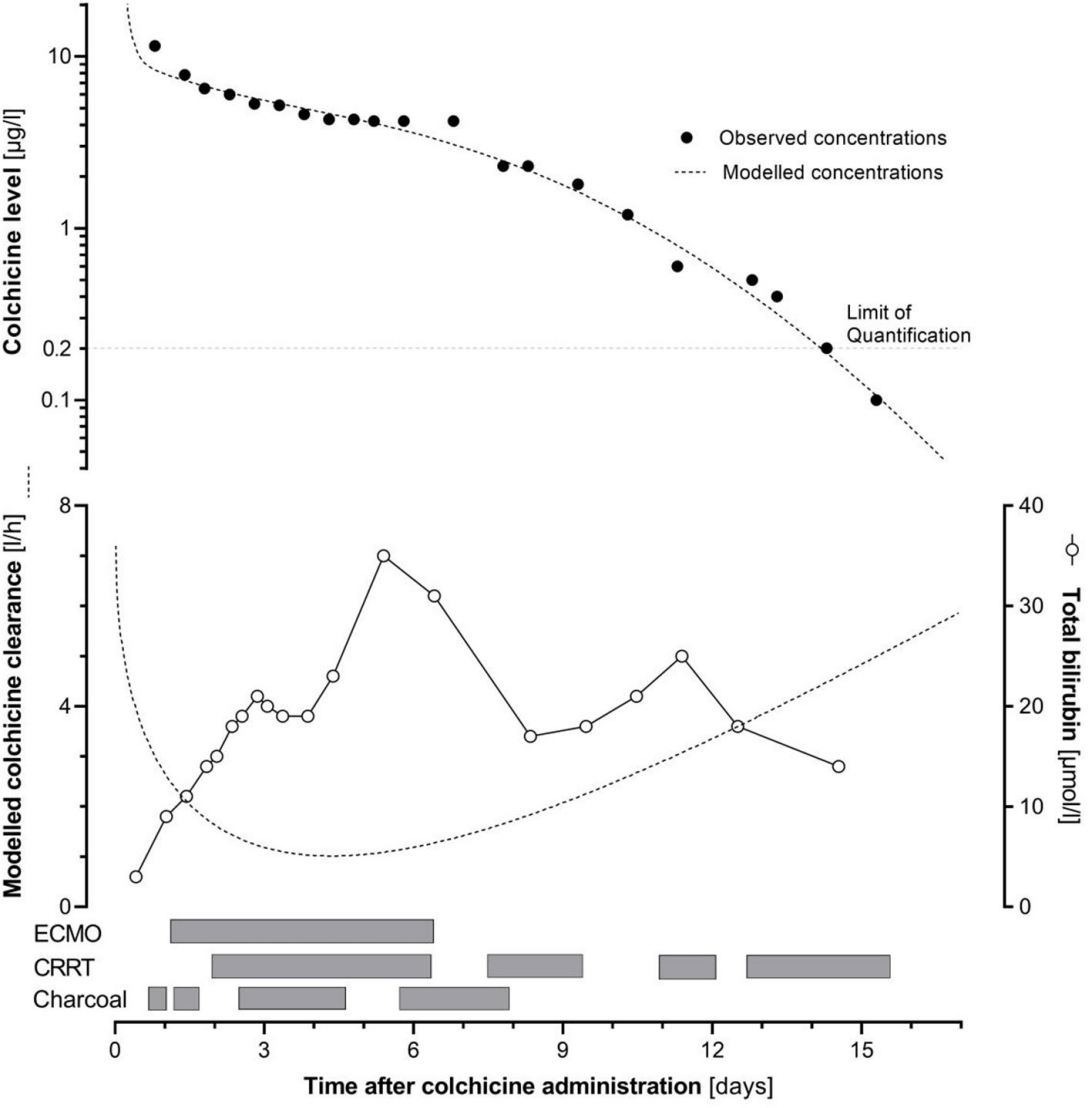
Upper panel: time course of colchicine concentration (points: observed and line: modeled concentrations), in relation with the administration of activated charcoal, renal replacement therapy (CRRT), and extracorporeal membrane oxygenation (ECMO). Lower panel: time course of total bilirubin concentration (points) and of modeled colchicine clearance (line).

We followed colchicine blood levels (*n* = 25) until clear clinical improvement (Figure 1). Values lower than the limit of quantification of the analytic method (0.2 μg/l) were measured from day 16 onwards. We observed 3 successive phases in the time-course of colchicine concentrations. An initial rapid exponential decay (days 1–2) was followed by a marked reduction in the slope down to a plateau (from day 2 to 7), and then the return of a progressively exponential decay, grossly similar to the initial phase.

## Toxicokinetic Analysis

We outlined a pharmacokinetic analysis using Microsoft Excel (Microsoft Corporation. Microsoft Excel 2018), based on a 2-compartment model described in healthy volunteers, with initial clearance and volumes corrected for body surface area of the patient (1). We found a *U*-shaped third degree polynomial equation (y = a + bx + cx^2^ + d sqrt(x)) appropriate to describe adequately the evolution of colchicine clearance over time. We sequentially included charcoal administration, renal replacement therapy, ECMO support (as categorical variables), creatinine concentrations, bilirubin concentrations, and thromboplastin times (continuous variables) as regressor factors impacting the clearance, without improvement of prediction of the observed colchicine concentrations. We show on Figure 1 the modeled colchicine concentrations, as well as the evolution of the colchicine clearance over time, according to our model. The evolution of the observed concentrations and the assumed colchicine clearance seem to be mainly driven by the hepatic function. A precise evaluation of liver function remains challenging, as there is no clear biomarker known to reflect accurately the extent of hepatic impairment, thus preventing its modeling in our patient.

## Discussion

Colchicine is a potent anti-inflammatory drug used in various indications as gout or Familial Mediterranean Fever. Its off-label use may include conditions as different as pericarditis, amyloidosis, and Behçet's disease (2, 3). Colchicine is known for a narrow therapeutic index. Hence, toxicity can be associated with exposures close to those obtained under usual therapeutic doses. While recommended pediatric daily doses range from 0.03 to 0.07 mg/kg, the ingestion of doses lower than 0.5 mg/kg usually result in minor toxicities (gastrointestinal and coagulation disorders), whereas the ingestion of doses above 0.5 mg/kg are associated with major toxicities (multiorgan failure, bone marrow aplasia) with reported mortality rates from 10 to 80% (4). Patients with early hemodynamic collapse have a particularly poor prognosis. Exceptional cases with cardiogenic shock and fatal outcomes have even been reported after ingested amounts below 0.5 mg/kg (5).

After oral intake, colchicine is rapidly absorbed, with serum peak levels occurring within 0.5–3 h (6). Its bioavailability is limited to 25–50%, due to significant first pass hepatic metabolism. Colchicine distributes readily into all tissues, where it binds to intracellular elements, in line with a large distribution volume (5–8 l/kg) (7). Elimination occurs mainly through liver metabolism, involving cytochrome P450 3A4 (CYP3A4), glucuronidation, P-glycoprotein transport (P-gp), and biliary excretion, with a significant enterohepatic recirculation (8, 9). Renal clearance only accounts for 10–20% of colchicine removal. The mean elimination half-life of oral colchicine is 4.4–16 h after therapeutic doses but may reach 11–32 h in poisoned patients (2, 9). Colchicine is susceptible to drug interactions, especially through inhibition of CYP3A4 or P-gp, which may enhance its toxicity (2).

The clinical presentation of acute colchicine poisoning is divided into three overlapping stages. The first stage (0–24 h post-ingestion) presents with peripheral leukocytosis and gastrointestinal symptoms resulting in hypovolemic shock. The second stage (1–7 days post-ingestion) is characterized by multi-organ dysfunction including bone marrow suppression with pancytopenia, hemolytic anemia, hepatic failure, renal failure, respiratory distress syndrome, cardiac arrhythmias, encephalopathy and brain edema, neuromuscular disturbances and myopathy, disseminated intravascular coagulation, and metabolic derangements such as metabolic acidosis, hypokalemia, hyponatremia, hypocalcemia, hypo- or hyperglycemia and hypophosphatemia. In surviving patients, the third stage (7–21 days post ingestion) shows recovery of multi-organ dysfunction, transient alopecia, and rebound leukocytosis. Death generally results from cardiovascular collapse during the first 48 h, or infection during the first 7 days (3).

Our patient ingested a potentially lethal colchicine overdose and experienced the classical signs of severe colchicine poisoning. She developed early cardiogenic shock, possibly favored by her recent cardiac surgery, followed by all toxic complications known for this agent. The mechanism of occurrence of this accident conveys a severe warning about the attention required during the prescription of narrow therapeutic margin, rarely used drugs, for which misunderstanding or inattention may result in dreadful consequences. At that time there was no exaggerated dose detection system in the electronic patient record. Since then, dosing alerts have been implemented on the prescription software to help preventing overdose in pediatrics.

Treatment of colchicine poisoning is mainly supportive. Experimental treatment with colchicine-specific antibody fragments has been successfully reported 25 years ago, but is still not routinely available (10). Gastric lavage is generally not recommended, but can be considered early after ingestion, in view of the potential severity of colchicine poisoning. Repeated doses of activated charcoal should be administered, even in cases of late presentation, as it may enhance elimination by interrupting the enterohepatic recirculation of colchicine. Extracorporeal elimination (hemodialysis and hemoperfusion) is ineffective due to the large distribution volume of colchicine. Plasma exchange treatment has been tried in cases of colchicine overdose with fatal outcomes (11, 12). Lipid emulsion therapy has not been assessed clinically for colchicine, however the chemical properties of colchicine make it an unlikely option (13). In severe situations, early supportive treatments should also be considered. G-CSF may be used to stimulate regeneration of suppressed bone marrow, but should be stopped early to avoid extramedullary hematopoiesis, especially in children. ECMO is emerging as a useful treatment modality for patients with severe poisoning (14). Nevertheless, thrombocytopenia and disseminated intravascular coagulation are common in significant colchicine poisoning and greatly increase the risk of bleeding complications. Owing to the poor prognosis of severe cases, the use of this treatment modality is still a matter of debate for colchicine poisoning (15, 16).

Considering the above literature, our patient received repeated administrations of activated charcoal for 8 days, and G-CSF for 3 days. Gastric lavage was not performed as the patient was admitted at the PICU only 14 h after ingestion. Plasma exchange treatment was not attempted considering the low level of evidence in the literature for this treatment. The patient received ECMO for 7 days because of refractory cardiogenic shock, and mechanical ventilation for 19 days.

Pharmacokinetic studies of colchicine are available at therapeutic doses for healthy adults and elderly patients (1, 17–19). A toxicokinetic analysis of overdose in adults revealed significant alterations in colchicine pharmacokinetic parameters with a larger apparent volume of distribution (21 l/kg), a longer elimination half-life and a higher fraction of the dose cleared by the kidney (30%) (20). Pediatric data are scarce for both therapeutic and toxic doses.

In our patient, colchicine blood levels were thoroughly followed (*n* = 25 samples), revealing three successive phases (Figure 1). An initial exponential decay corresponded to the described pharmacokinetic properties of the drug, before the occurrence of multi-organ failure. Thereafter, a plateau in colchicine concentrations over 5 days indicated a minimal elimination during this time period. This plateau occurred during the phase of multi-organ failure and impairment of the eliminating organs, especially the liver. Delayed absorption is unlikely to play a role in this plateau, since it occurred much later than the expected end of the absorption phase and concomitantly to the administration of large doses of activated charcoal. Reviewing case reports of severe colchicine poisoning in adults, we identified a similar pattern with a concentration plateau (10, 21). This temporary lack of elimination is probably a consequence of the impairment of elimination organs, but the resulting persistent exposure also likely contributes to a vicious cycle, explaining the poor prognosis of severe colchicine poisoning. During the plateau phase, colchicine concentrations remained stable irrespective of the administration of activated charcoal, renal replacement therapy or ECMO. Figure 1 illustrates this lack of temporal relationship. Thus, renal replacement therapy added no significant clearance of colchicine, nor did charcoal improve elimination during this phase. This may result from the shutting down of the enterohepatic circulation, likely caused by liver impairment. In parallel with the resolution of most organ dysfunctions, including liver, an exponential decay was restored.

## Conclusion

To the best of our knowledge, we report here the first pediatric case of severe colchicine poisoning (potentially lethal dose) surviving persistent cardiogenic shock and multi-organ failure with the support of ECMO therapy.

In the context of multi-organ failure, colchicine showed a marked slowing of its elimination leading to a concentration plateau, as a result of hepatic impairment. Although clearly indicated, neither renal replacement therapy, nor activated charcoal administration enabled a significant colchicine clearance during this phase.

This case highlights the significant alterations in colchicine pharmacokinetic parameters in the context of severe intoxication and the importance of early supportive care for the management of such critical situations.

## Data Availability Statement

The original contributions presented in the study are included in the article/Supplementary Material, further inquiries can be directed to the corresponding author/s.

## Ethics Statement

Ethical review and approval was not required for the study on human participants in accordance with the local legislation and institutional requirements. Written informed consent to participate in this study was provided by the participants' legal guardian/next of kin.

## Author Contributions

MPM and SV managed the patient and drafted the manuscript. TF, VA, JN, and M-HP managed the patient, contributed to data collection, and revised the manuscript. SP, TB, and HC performed the pharmacokinetic analysis and drafted and revised the manuscript. DL managed the patient, contributed to data collection, revised the manuscript, and gave final approval of the version to be submitted. All authors have read and approved the final manuscript.

## Conflict of Interest

The authors declare that the research was conducted in the absence of any commercial or financial relationships that could be construed as a potential conflict of interest.
