# Supplementary figures and images for: Case Report: Colchicine Toxicokinetic Analysis in a Poisoned Child Requiring Extracorporeal Life Support
Source: Front Pediatr. 2021 Apr 7;9:658347. doi: 10.3389/fped.2021.658347 (PMC8058177; doi:10.3389/fped.2021.658347)

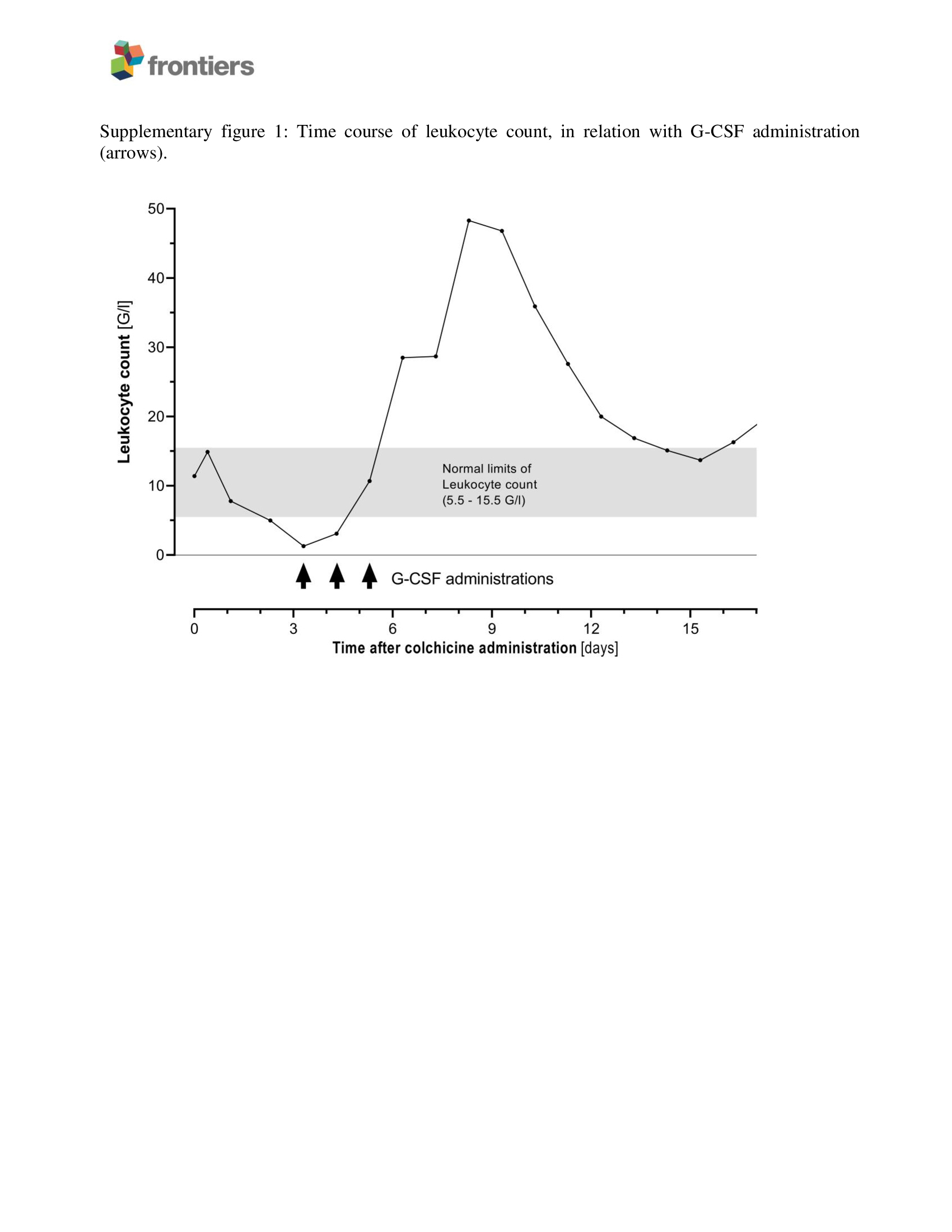

Supplement: Supplementary file 1 [file Image_1.jpg]
